# Skin-Dwelling Bacteria Survive Preoperative Skin Preparation in Reconstruction Surgery

**DOI:** 10.3390/jcm14103417

**Published:** 2025-05-14

**Authors:** Hannah R. Duffy, Nicholas N. Ashton, Porter Stulce, Abbey Blair, Ryan Farnsworth, Laurel Ormiston, Alvin C. Kwok, Dustin L. Williams

**Affiliations:** 1 Department of Orthopaedics, University of Utah, Salt Lake City, UT 84112, USA; hannah.duffy@utah.edu (H.R.D.); n.ashton@utah.edu (N.N.A.); u1270386@utah.edu (P.S.); abbey.blair@hci.utah.edu (A.B.); u1190395@utah.edu (R.F.); 2 Department of Biomedical Engineering, University of Utah, Salt Lake City, UT 84112, USA; 3 Department of Surgery, Division of Plastic Surgery, University of Utah Health, Salt Lake City, UT 84112, USAalvin.kwok@hsc.utah.edu (A.C.K.); 4 Department of Pathology, University of Utah, Salt Lake City, UT 84112, USA; 5 Department of Physical Medicine and Rehabilitation, Uniformed Services University of the Health Sciences, Bethesda, MD 20814, USA

**Keywords:** preoperative skin preparation, surgical site infection, skin microbiome, bacterial sampling

## Abstract

**Background/Objectives:** Accurately determining the bacterial bioburden that survives preoperative skin preparation (PSP) is critical in understanding PSP efficacy and its limitations. Clinical PSP approval relies on a bacterial sampling method described in the American Society for Testing and Materials (ASTM) standard E1173-15. Though common, this technique may overlook deep-dwelling skin bacteria. The objective of this study was to test the hypothesis that deep-dwelling skin flora would survive PSP, and more growth would be detected using a destructive sampling method compared with ASTM E1173-15. **Methods:** Twelve female participants with a scheduled deep inferior epigastric perforator (DIEP) artery flap procedure at the Huntsman Cancer Institute in Salt Lake City, UT, were enrolled between January and August 2024. PSP was performed using three 26 mL ChloraPrep applicators (2% CHG), and excess tissue was collected. Bacteria in the skin were quantified using a destructive sampling method and ASTM E1173-15, and bioburden outcomes were compared. Two participants were excluded from the quantitative analysis. **Results**: Bacteria survived PSP in every participant. A greater diversity and more bacteria were quantified with destructive sampling than ASTM E1173-15 (*p* < 0.01). Generally, anaerobic bioburden values were higher than aerobic bioburden values. Higher bioburden correlated with processing more skin from a participant. Genotypic identification of select isolates identified *Staphylococcus epidermidis* and *Cutibacterium acnes* (formerly known as *Propionibacterium acnes*) as surviving bacteria, among others. Immunofluorescence revealed bacteria in all skin layers. No participant exhibited clinical signs of infection in the abdominal region. Human data corroborated previous porcine data collected using destructive skin sampling after PSP. **Conclusions**: Clinical PSP application does not create a sterile field. Destructive skin sampling techniques may be more effective than ASTM E1173-15 at resolving bacterial PSP survivors contributing to SSI risk.

## 1. Introduction

Since the adoption of preoperative skin preparation (PSP) and modern surgical techniques, infection rates have plummeted to single digits, yet the efficacy of clinical PSP may be overstated [1,2,3]. PSP-surviving microorganisms colonize surgical sites, leaving 55–90% of surgical wounds contaminated upon closure [4,5]. Bacterial colonizers principally originate from deeper skin layers, out of antiseptic reach, and may replicate in the surgical site. Surviving bacteria account for 70–95% of surgical site infections (SSIs) [6]. Thus, endogenous microflora is the most significant contributor to SSIs, exceeding contaminants from room air, instruments, or surgical personnel [6,7,8,9]. Consequences of SSIs are severe: SSIs increase patient morbidity and mortality and cost the United States (US) healthcare system up to 10 billion USD annually [1,7]. Tissue quantification techniques that thoroughly detect microbial survival following PSP are critical in determining PSP efficacy and addressing SSIs.

Histological and quantification evidence indicates that bacteria survive PSP. Gram staining, scanning electron microscopy, and fluorescence imaging have shown that bacteria live throughout the skin’s layers, concentrated along hair follicle tracts and pilosebaceous glands [10,11,12,13]. Since 1949, operating room contamination has been deemed largely preventable except for bacteria originating from the patient microbiota; chemical skin sterilization was considered “impossible” [14,15]. In the operating room, surgical instruments carry up to 4.4 × 10^3^ colony forming units (CFU) after use [16], most patients have positive cultures from at least one swabbed location before wound closure [17], and pedicle screws handled in the sterile field culture 10^5^ to 10^7^ CFU per screw [18]. Notwithstanding PSP and aseptic techniques, more than 70 years of data show viable bacteria living deep in skin layers.

Resolving deeper-dwelling microbes using today’s test methods is challenging. The industry relies on the American Society for Testing Materials (ASTM) standard E1173-15, *Standard Test Method for Evaluation of Preoperative*, *Precatheterization*, *or Preinjection Skin Preparations*, for testing chemical antiseptics [19]. In 1994, 59 Federal Register 31402 was released by the Food and Drug Administration (FDA), promoting ASTM E1173-15. This standard is referred to as the cup scrub method as it comprises the following steps: (1) filling a sterile cylinder (the cup) on the skin with solution, (2) suspending skin bacteria into the liquid by scrubbing with a rubber spatula, (3) culturing the suspension. While the cup scrub method is common, it fails to resolve deep-dwelling microbes. Researchers estimate that scrubbing accounts for 4–85% of the total microflora [11,20,21,22]. For example, skin scrubbing produced an average bioburden of 5.1 × 10^3^ CFU/cm^2^ compared with 3.0 × 10^4^ CFU/cm^2^ from a small excision [11]. Similarly, the calculated total skin bioburden was 5 × 10^4^ CFU/cm^2^ for skin scraping, but 10^6^ CFU/cm^2^ for a biopsy [21]. These investigations indicate that scrubbing methods may not detect bacterial PSP survivors from deeper skin layers [21,22].

In contrast to scrubbing methods like the cup scrub method, destructive methods excise and destroy the skin where PSP is applied and account for deeper-dwelling organisms. Previously, we used full-thickness tissue homogenization—the tissue blend method—in a Yorkshire/Landrace hybrid pig model to test the effectiveness of common clinical PSP [23,24]. Neither povidone-iodine nor chlorhexidine gluconate (CHG) produced the required 2–3 log_10_ reduction proposed by the FDA [23]. When we compared the cup scrub method with the tissue blend method following a 4% CHG PSP, the tissue blend method resolved more than 100× the bacteria than the cup scrub method/cm^2^ of pig skin [24]. The tissue blend method is advantageous because it uses large surface areas, and PSP can be rapidly screened for effectiveness. In clinical practice, destructive methods are usually limited to a biopsy punch. However, extra tissue may be available in reconstructive surgeries, such as deep inferior epigastric perforator (DIEP) artery flap procedures.

Previous studies quantifying the bacterial survivors after PSP focused on the bacteria present on surgical tools or have insufficiently addressed deep-dwelling microorganisms [4,16,17,18]. In this study, we applied the cup scrub and tissue blend methods to discarded skin from DIEP surgeries. The primary objective was to quantify PSP-surviving microbes across a large surface area and depth of human skin. We hypothesized that skin flora would survive PSP, and more growth would be detected using the tissue blend method than the cup scrub method. The secondary objective was to determine if the post-PSP bacterial profiles in human skin paralleled those in pig skin. We hypothesized that human skin would have similar quantities and types of bacterial survivors following PSP as in pigs.

## 2. Materials and Methods

### 2.1. Recruitment

Following approved Institutional Review Board protocol 00161032 (approved on 11 October 2023) at the University of Utah, 12 participants from the Huntsman Cancer Institute in Salt Lake City, UT, were recruited between January and August 2024 (2 were excluded from quantitative and statistical analysis based on using an alternate PSP or systemic antibiotic use disclosed after collection, see Section 2.2 below). Participants were English-speaking adults (18+) with a scheduled DIEP procedure at the Huntsman Cancer Institute. Patients in a vulnerable population including pregnant patients, prisoners, children, or disabled individuals were excluded from participation.

All subjects provided written informed consent to participate in this observational study. Gender was participant-identified. Individuals who were in vulnerable populations, taking systemic antibiotics at the time, or undergoing cancer treatments were excluded. Treatment that occurred >14 days before surgery was allowed. No compensation was provided.

For sample size determination, we assumed the same effect size as a previous animal study comparing the tissue method with the cup scrub method [24]. We reported means of 3.24 and 1.05 log_10_ CFU/cm^2^ for the tissue blend and cup scrub methods, respectively. Standard deviations (disregarding data clustering) were 1.10 and 1.11 log_10_ CFU/cm^2^ for the tissue blend and cup scrub methods, respectively [24]. We required 6 participants per group to detect this effect size with 80% power using a two-sided comparison (alpha = 0.05). We recruited more than 6 participants per group to allow flexibility in our sample size assumptions. When possible, we further increased the sample size by collecting data from multiple sites using each method type (tissue blend and cup scrub) per participant. This additional data collection increased the statistical power to greater than 80%.

### 2.2. Surgery and Skin Collection

**Figure 1:**
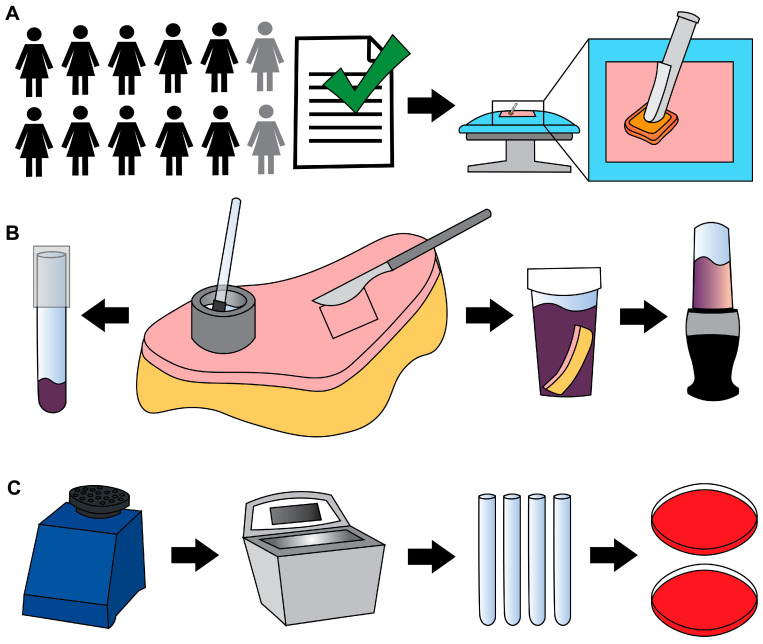
Methods schematic. (**A**) Informed consent of 12 female participants. Ten were included in the quantitative analysis. Before surgery, each participant’s skin was treated with PSP. (**B**) Participant tissue was processed using the cup scrub method using a stainless-steel cylinder (the cup), a small rubber spatula, and a glass tube (left arrow). The tissue blend method involved using a scalpel to cut full-thickness skin and a blender to homogenize the tissue (right arrows). CHG was neutralized using D/E broth. (**C**) Each sample was vortexed, sonicated, serially diluted, and plated on Columbia blood agar in duplicate, then incubated under aerobic or anaerobic conditions.

PSP was performed using three 26 mL BD ChloraPrep Hi-Lite applicators (2% CHG in a solution of 70% isopropyl alcohol) (Figure 1A). Cefazolin (~2 g) was prophylactically administered intravenously within 30 min of incision. Antimicrobial washes or wipes were not prohibited.

Excess abdominal skin tissue was collected aseptically, concurrent with surgery. Specimen(s) were placed in 1 or 2 sterile containers and transported to the Bone and Biofilm Research Lab (BBRL) for immediate processing (a 10-min walk).

Samples were collected from two individuals who were excluded from the quantitative and statistical analysis. The first exclusion occurred after the participant received a povidone-iodine PSP instead of a CHG PSP. As the regular surgical protocol at the Huntsman Cancer Institute consists of a CHG PSP, except in the case of allergy or sensitization, we excluded the numerical data for this individual to maintain PSP consistency across the participant pool. For the second exclusion, it was only after tissue collection was performed that we learned the participant had received systemic antibiotic treatment. This participant was not taking systemic antibiotics upon consent and, thus, was enrolled. We excluded this participant’s data because systemic antibiotics could have influenced bioburden outcomes and, thus, data analysis. We reported the outcomes of these participants only generally.

### 2.3. Processing

The skin was aseptically transferred to a sterile surface. Areas were designated for cup scrub and tissue blend samples. The cup scrub method was performed using up to 5 sterile, custom-made stainless-steel cylindrical cups (1 ½ in. outer diameter × ¼ in. wall thickness × 1 in.) as previously described [24]. Cup scrub sampling solution was mixed following ASTM E1173-15 by dissolving 0.4 g KH_2_PO_4_ (1048711000, MilliporeSigma, Burlington, MA, USA), 10.1 g Na_2_HPO_4_ (ACM7558794, Alfa Chemistry Materials, Holbrook, NY, USA), and 1.0 g of Triton X-100 (108643, MilliporeSigma) in 1 L of distilled water, then autoclaved [19]. With a cup held in place on the skin’s surface, 3 mL of prepared solution were pipetted into the cup (Figure 1B). A sterile rubber spatula (Cole-Parmer, Vernon Hills, IL, USA) was used to scrub the skin within the cup (~1 min). One mL of solution from the cup was pipetted into a sterile tube containing 1 mL of Dey–Engley (D/E) neutralizing broth (D3435, MilliporeSigma). Tubes were vortexed (1 min) and sonicated (10 min) (Figure 1C).

The tissue blend method was performed by cutting 4 cm × 4 cm full-thickness samples, leaving ~1–2 cm of subcutaneous fat. Tissue pieces were placed individually into sterile sample containers (2767M1, Medicus Health, Kentwood, MI, USA) containing 50 mL of D/E broth. When the skin’s geometry did not allow a 4 cm × 4 cm shape, images of the pieces were collected, and the surface area was determined using ImageJ (version 1.54g, National Institutes of Health). Where possible, sections were cut to areas approaching 16 cm^2^. Tissue sections used to collect cup scrub data were not used to collect tissue blend data, and vice versa.

Blender cups and blades (Ninja QB3001SS Fit Compact, SharkNinja, Needham, MA, USA) were cold sterilized using 200-proof ethanol as described previously [23,24]. Blenders were run with ethanol (~15 s), then rinsed and blended with sterile deionized water (~15 s).

Full-thickness tissue samples and D/E broth were transferred to a sterile blender cup and blended (45 s), vortexed (1 min), and sonicated (10 min) as previously described [24]. Blending was performed without tissue to confirm that the processing technique was contamination free [23,24].

Four hundred µL of each sample mixture were plated on Columbia blood agar (A16, Hardy Diagnostics, Sandy, UT, USA) to make a 0-dilution plate. Bioburden was quantified as previously described [23]. Plates were incubated under aerobic and anaerobic conditions using Anaerogen packs (AN25US/AN35US, Hardy Diagnostics) for 48 ± 4 h and 72 h to 5 days, respectively, at 37 °C in a jacketed incubator. Colony counts constituted bioburden (CFU/cm^2^).

A representative colony of each distinct morphology from the individual study groups was isolated with a sterile loop, cultured on agar, cataloged, and cryopreserved (−80 °C) as previously described [23]. Isolates were classified by morphology, color, and Gram stain to determine biodiversity. Nelson Labs genotyped ten unique species using Organism ID: Genotypic, MicroSeq w/Gram stain.

### 2.4. Additional Analyses: Clinical Outcome, Surface Area and Time

A retrospective chart review was performed to record surgical outcomes (including infection) at least 90 days after surgery. We also analyzed the number of samples processed per participant (correlated to increased surface area) compared with bioburden. Finally, we investigated the impact of surgical and processing (collection to incubation) times (to the nearest half hour) on bioburden.

### 2.5. Statistical Analysis

Up to five samples of the same method type (cup scrub or tissue blend) from the same participant introduced data clustering across our participant population. Therefore, we analyzed the data using a mixed-effects linear regression, also known as a multilevel model, using a restricted maximum likelihood (REML). This process accounted for multiple samples within a single participant’s tissue. We used an REML fitting algorithm in order to obtain *p* values based on the t distribution, rather than base significance tests on the z distribution used by other fitting algorithms. This makes the REML model more correct for small sample sizes.

In our model, the experimental condition (cup scrub or tissue blend) was a fixed effect, and the participant was a random effect. Average bioburden values were reported as the mean ± the standard error. All statistical results were obtained using Stata statistical software (version 18.0, StataCorp LLC, College Stata, TX, USA). All reported *p* values are from a two-sided comparison, where statistical significance was set at *p* < 0.05.

### 2.6. Histology

ARUP Laboratories performed Hematoxylin and Eosin (H&E) staining. Four participant skin samples were selected for immunofluorescence (IF) analysis based on features present on the H&E stains. IF staining was performed using a Gram-Positive Bacteria Lipoteichoic acid (LTA) Monoclonal Mouse Antibody (primary, G43J, ThermoFisher Scientific) and a Goat Anti-Mouse IgG Alexa Fluor 488 Polyclonal antibody (secondary, SouthernBiotech, Birmingham, AL, USA). The blocking buffer consisted of 1% Bovine albumin (Millipore Sigma), 0.1% Tween 20 (Sigma-Aldrich, St. Louis, MO, USA), and 0.1% Triton-X 100 (Sigma-Aldrich) diluted in phosphate-buffered saline. Visualization occurred using inverted microscopes: a Nikon Eclipse E600 (Nikon, Minato City, Tokyo, Japan) for H&E and Leica DMi8 (Leica, Wetzlar, Germany) for IF.

### 2.7. Comparison to Previous Work

We compared the bioburden outcomes of this study to those of previous animal work [23,24].

## 3. Results

Participants were females aged 35 to 65 (mean = 48.6 ± 8.1 years). No participant exhibited clinical signs of infection in the abdominal region. Antibiotics were administered to two participants prophylactically after surgery for issues unrelated to the flap removal.

**Figure 2:**
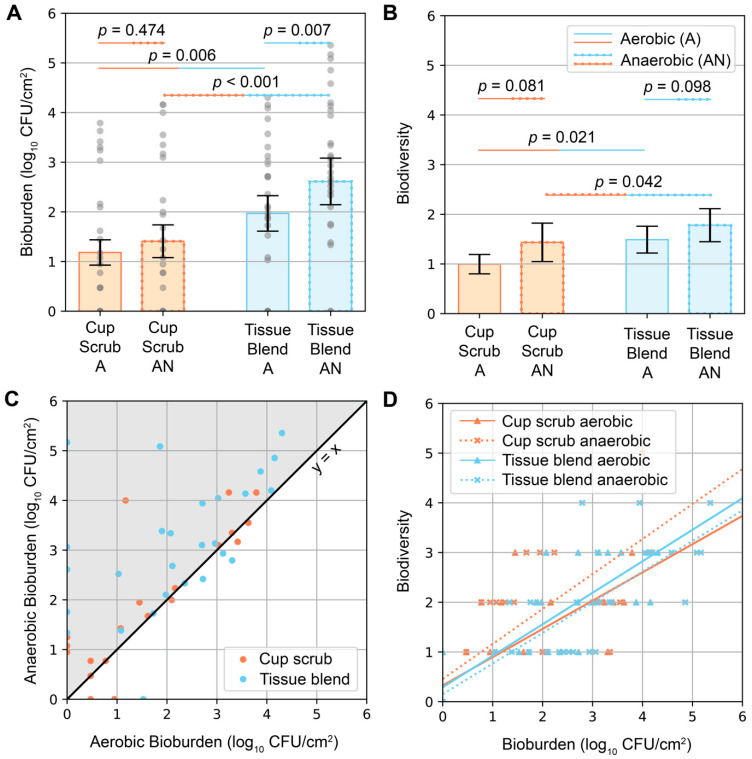
Aerobic and anaerobic bioburden quantities and biodiversity following the cup scrub and tissue blend methods. “A” = aerobic conditions. “AN” = anaerobic conditions. (**A**) Bioburden (log_10_ CFU/cm^2^) differentiated by processing method and incubation environment. Orange bars represent the cup scrub method. Blue bars represent the tissue blend method. Gray circles represent pooled data points across all participants. Error bars show the standard error. (**B**) Biodiversity as determined by colony types using morphology and Gram stain. Orange bars represent the cup scrub method. Blue bars represent the tissue blend method. Error bars show the standard error. (**C**) Anaerobic and aerobic bioburden for each sample were plotted against each other as (aerobic bioburden, anaerobic bioburden). The black line is x = y. Most samples had higher anaerobic bioburden than aerobic bioburden (gray area). (**D**) Biodiversity correlated positively with bioburden across all sample groups. The linear regression trendlines were y = 0.57x + 0.33 (cup scrub aerobic), y = 0.70x + 0.46 (cup scrub anaerobic), y = 0.63x + 0.29 (tissue blend aerobic), and y = 0.62x + 0.15 (tissue blend anerobic).

Bacteria were detected in the PSP-prepared skin of every participant. Bioburden quantification values ranged from below detectable limits (noted with 0 log_10_ CFU/cm^2^ in Figure 2) to 5.36 log_10_ CFU/cm^2^. The detection limits for the cup scrub and tissue blend methods were 0.47 and 0.89 log_10_ CFU/cm^2^, respectively. Variability was observed across participants and sampling methods. Eight out of ten participants had aerobic growth using the cup scrub method. Eight out of ten participants had aerobic growth using the tissue blend method, although these participants differed from those with positive cultures using the cup scrub method. A negative culture result using one method was not predictive of the other. Some samples with negative aerobic cultures exhibited growth on the anaerobically cultured plates. Infrequently, a cup scrub sample exhibited substantial growth, while the tissue blend sample taken from the skin immediately adjacent had little to no detectable growth. Yet, tissue blend samples generally exhibited more growth than cup scrub samples.

Higher bioburden was observed in tissue blend samples compared with cup scrub samples (Figure 2A). Cup scrub samples had 1.18 ± 0.25 (mean ± standard error) and 1.41 ± 0.33 log_10_ CFU/cm^2^ for aerobically and anaerobically grown cultures, respectively (*p* = 0.474). Tissue blend samples had 1.97 ± 0.36 and 2.61 ± 0.47 log_10_ CFU/cm^2^ for aerobically and anaerobically grown cultures, respectively (*p* = 0.007). Data point clusters increased from the cup scrub aerobic bioburden to the tissue blend anaerobic bioburden. On average, there were 0.79 log_10_ more CFU/cm^2^ cultured aerobically from tissue blend samples than from cup scrub samples (*p* = 0.006). Similarly, 1.20 log_10_ more CFU/cm^2^ cultured anaerobically from tissue-blended samples than cup scrub samples (*p* < 0.001).

Similar trends were observed when considering biodiversity (Figure 2B). Per 400 µL of plated solution, all samples had between 0 and 5 different bacterial types. The cup scrub method resulted in 1.00 ± 0.20 (mean ± standard error) and 1.43 ± 0.39 bacterial types for aerobic and anaerobic samples, respectively. The tissue blend method resulted in 1.49 ± 0.27 and 1.78 ± 0.33 bacterial types for aerobic and anaerobic samples, respectively. The difference between the cup scrub and the tissue blend methods was significant (*p* = 0.021 for aerobic and *p* = 0.042 for anaerobic).

Anaerobic bioburden values were higher than aerobic bioburden values for the same sample (Figure 2C). Biodiversity correlated positively with bioburden (Figure 2D); the more bacteria we observed, the more likely we were to observe different bacterial types.

Samples cultured from a participant who received a PSP containing povidone-iodine resulted in bioburden values similar to those who received a CHG PSP. In contrast, the individual who received systemic antibiotics had calculated bioburdens that approached 0 CFU/cm^2^ or were below detectable limits. Most plates from this individual had no growth. This result prompted the investigation into the clinical case which exposed the antibiotic dosage.

**Figure 3:**
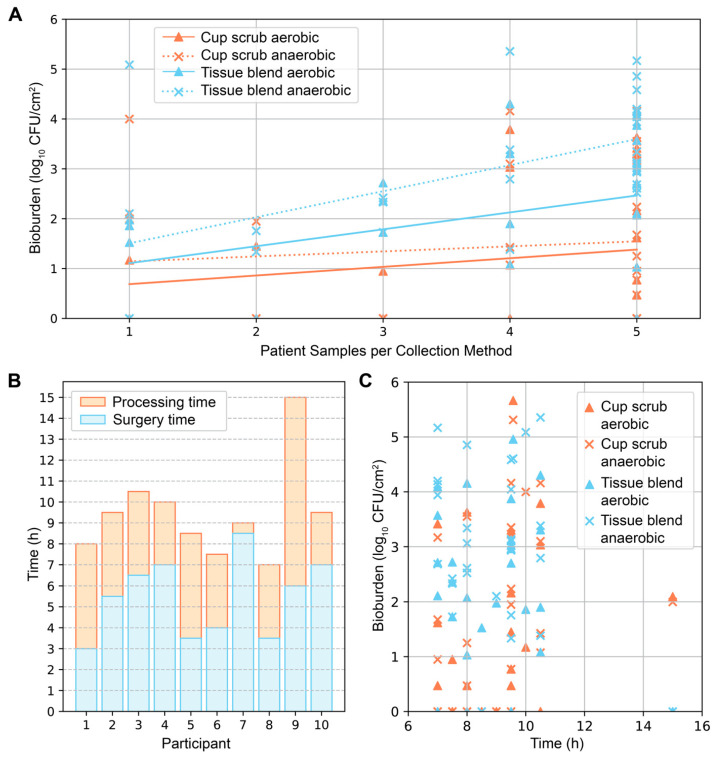
Additional analyses of surface area and time. (**A**) Bioburden (log_10_ CFU/cm^2^) plotted against the number of skin samples collected per patient differentiated by sampling technique and incubation environment. The number of skin samples (1–5) correlated with increased surface area for each sampling technique by approximately 5 cm^2^ for cup scrub samples and 16 cm^2^ for tissue blend samples. Positive trendlines were observed: y = 0.17x + 0.51 (cup scrub aerobic), y = 0.10x + 1.04 (cup scrub anaerobic), y = 0.34x + 0.77 (tissue blend aerobic), and y = 0.52x + 0.98 (tissue blend anerobic). (**B**) Time from surgery start to processing end for each participant. Surgery time was calculated from surgery start until estimated tissue removal. Processing time was calculated using the estimated tissue removal time to incubation time. Transport and laboratory processing time varied. (**C**) No correlation was observed between the total time from surgery start to processing end and bioburden.

Secondary investigations of surface area and time produced varying results. In general, the more skin samples we obtained from a participant, the greater the average bioburden (Figure 3A). This pattern was most notable in samples that were tissue-blended and cultured anaerobically. The time from surgery start to tissue removal, and from removal to completion of sample processing varied from participant to participant (Figure 3B). These differences depended on the type of surgery (unilateral or bilateral), surgical complications, the number of samples collected, and varying processing duration. From start to finish, the surgical and sample processing times ranged from 7 to 15 h (mean = 9.5 ± 2.2 h). The average surgical time and processing times were 5.5 ± 1.9 h (mean ± standard deviation) and 4.0 ± 2.2 h, respectively. Plotting the total surgical and processing times against bioburden did not elucidate a distinguishable pattern; bioburden levels of all types were found across varying times (Figure 3C).

Of the 116 representative isolates analyzed, nearly all (96.6%) were Gram-positive, with 57.8% cocci and 42.2% rods. Most species grew aerobically and anaerobically. We identified the following unique species (not representative): *Staphylococcus epidermidis*, *Cutibacterium acnes*, *Bacillus toyonensis*, *Staphylococcus lugdunensis*, *Lysinibacillus xylanilyticus*, *Cupriavidus pauculus*, *Chryseobacterium massiliae*, *Enterococcus casseliflavus*, *Staphylococcus lugdunensis*, and *Stenotrophomonas pavanii*.

**Figure 4:**
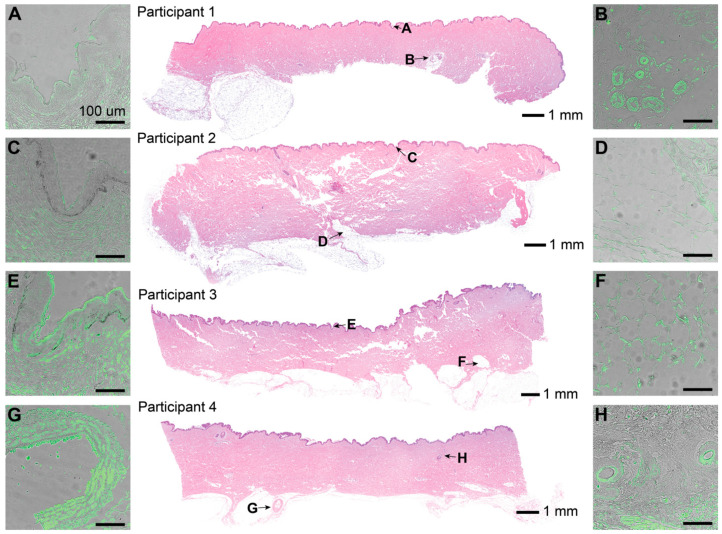
Representative histological sections from the skin of 4 participants treated with PSP (CHG or povidone-iodine). The central column shows skin sections stained with H&E (4× magnification, processed using Adobe Lightroom, version 4.3), letters corresponding to areas of the skin cross-section, and individual scale bars. The left and right columns show 20× magnified raw images of the skin sections stained with IF targeting LTA as an antigen using 250 ms of exposure, a gain of 4, and 55% light intensity. IF was overlayed with the background image of the same slide using 10 ms exposure and 90% light intensity. Each scale bar for (**A**–**G**) represents 100 μm. (**A**) Stratum corneum from participant 1. (**B**) Sebaceous gland from participant 1. (**C**) Stratum corneum from participant 2. (**D**) Subcutaneous tissue from participant 2. (**E**) Stratum corneum from participant 3. (**F**) Subcutaneous tissue from participant 3. (**G**) Larger hair follicle (partially broken) from participant 4. (**H**) Two small hair follicles from participant 4.

H&E stains showed characteristic skin samples of the abdominal region (Figure 4). One sebaceous gland was observed in the skin of participant 1. Hair follicles were only observed in participant 4. IF corresponding to LTA, a characteristic component of Gram-positive bacterial cell walls, was observed in pockets scattered throughout all layers throughout the epidermis and dermis (Figure 4A–G). Fluorescence intensity varied across patients, sections, and locations, but was present in every slide stained. The signal was especially concentrated around features such as sebaceous glands and hair follicle shafts when present. Positive and negative controls were used to confirm the presence of bacteria.

**Figure 5:**
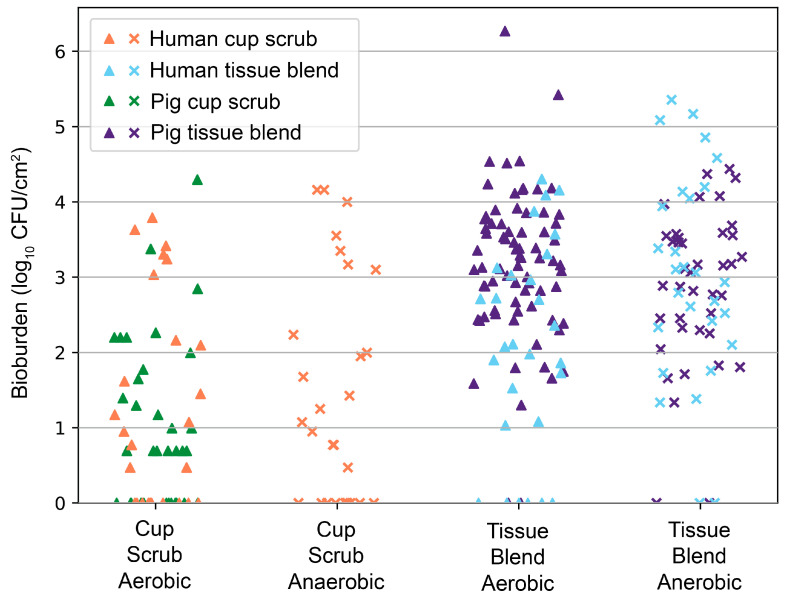
Overlay of human cup scrub and tissue blend bioburden following a CHG PSP with previously collected pig cup scrub and tissue blend bioburden (log_10_ CFU/cm^2^) from 2021 and 2022 [23,24]. Each point represents a unique sample collected from either a participant or an animal. Human cup scrub data are indicated in orange. Human tissue blend data are shown in blue. Pig cup scrub data are recorded in green. Pig tissue blend data are marked in indigo.

Data were consistent with our previous animal findings of CHG PSP efficacy [23,24]. An overlay of pig data correlated with the human skin outcomes (Figure 5). The difference between the cup scrub and tissue blend methods was significant in both pigs (*p* < 0.001, aerobic) and humans (*p* = 0.006, aerobic)

## 4. Discussion

PSP is a critical disinfection step before surgery, but quantification and histological data show that viable bacteria remain post PSP. Our hypothesis that skin flora would survive PSP and more bacteria would be detected with the tissue blend method was supported. Samples quantified using the tissue blend method consistently cultured more bacteria from all skin regions compared with the cup scrub method, underpinning the potential benefit of using a destructive skin sampling method to test PSP. Our secondary hypothesis was also supported as post-PSP bacterial profiles in human skin were similar to what we previously identified in pig skin [23].

The cup scrub method primarily considered surface-dwelling bacteria, while the tissue blend method considered all skin layers using full-thickness excisions. At most, the cup scrub rubber spatula could dislodge bacteria from a few cell layers deep. Thus, more than 10x the quantity of bioburden was detected using the tissue blend method compared with the cup scrub method for anaerobic cultures. Biodiversity results showed a less drastic difference between the two methods than bioburden. In positive cultures, only one or two species usually dominated, even if more types of bacteria were present. This finding suggested that bacterial communities are often found in pockets and not evenly distributed.

The presence of *C. acnes*, a common skin commensal and frequent SSI culprit of shoulder, breast, and spine surgery, may explain why anaerobic cultures were more plentiful and diverse than aerobic ones [25,26,27]. Because *C. acnes* grow best in a low-oxygen environment and were not visible in aerobic colony counts, we allowed the anaerobic plates to incubate for up to 5 days [28]. In the skin, anaerobic environments can be found in the hair follicles and glands, deep enough to be suspended in sebum. In contrast, some participants had matching aerobic and anaerobic counts, indicating that *C. acnes* were not necessarily colonizing all participants. These findings are supported by previous work [20,29].

The data correlating higher bioburden with more surface area suggest that operations with large surgical incisions introduce more bacteria into the surgical site than smaller incisions. Across a bacterial population that is randomly distributed, the likelihood of a direct hit with a surgical instrument to a highly colonized follicle increases with greater surface area transected. Additionally, surgeries with larger incisions often incorporate more biomaterials, e.g., sutures, that may become a nidus for bacterial replication. Biomaterials may reduce the infectious dose of bacteria by 10^3^ CFU/g [30]. While previous work has shown that biomaterials with antimicrobial coatings may be useful in minimizing this effect, infections persist [31]. These scenarios warrant future work to assess the benefits of antimicrobial biomaterials and minimally invasive laparoscopic or robotic surgery to decrease bioburden.

The culture results using the tissue blend method addresses the weaknesses of previous work by quantifying skin bioburden using large surface areas and full-thickness depths. In a comparison of abdominal colonization using a modified cup scrub method and a 1 cm^2^ excision, the cup scrub method produced 5.1 × 10^3^ (3.7 log_10_) CFU/cm^2^ compared to 3.0 × 10^4^ (4.5 log_10_) CFU/cm^2^ from the excision [11]. While these values are higher than what we detected from the individuals in this study, no PSP type or processing method was specified for the excisions. In a different group of patients, 14.5% of the skin biopsies cultured bacteria immediately following PSP preparation. This percentage is lower than our 100% positive detection rate; however, the study did not list surface area size or depth and, therefore, is not a true comparator [5]. The positive culture rate for the same group of patients rose to 55% using a cotton swab on the skin’s surface after wound closure, suggesting that bacteria may have initially survived below detectable limits and replicated in the surgical wound [5]. Similarly, 71% of open-heart surgery patients were culture-positive from a tissue swab [17]. Others have suggested that ~90% of implantation sites may be immediately colonized [4]. While swab or selective cultures produce valuable data, they may underreport bacterial survival [11]. The design of this study fully assesses PSP-surviving microbes by using large surface areas and accounting for microbes in all skin layers.

Patterns of fluorescence confirmed microbiological outcomes showing bacteria in all skin layers. The lack of skin features was likely due to the anatomical region we investigated (abdomen) and the participant population we recruited. This area is relatively hairless in females, and glands are sparser than regions with more hair and moisture. Despite fewer features, fluorescence pockets of varying intensity were observable across all slides and skin layers. Thus, the bioburden we reported may be a low-end estimate compared with areas with dense skin appendages. The nature of this study provides a basis for future work investigating the bioburden of various anatomical locations following PSP.

This small cohort study was principally designed as an observational investigation for hypothesis testing and, therefore, had limitations. Due to the nature of the DIEP flap surgery as a breast reconstruction procedure following mastectomy, we recruited from a female-identifying population. There are some variations between males and females stemming from physiological and anatomical differences between the sexes. Sweat, sebum, and hormone production levels in males and females as well as hair density and pH play a large role in the skin microbiome [32,33]. Though significant, sex is one of many factors including age, ethnicity, and location of residence that significantly impact our microbial communities [33]. Although we did not collect specific data on patient location of residence, participants were all receiving care in Salt Lake City, UT, further increasing participant similarity. Finally, immediate microbiological analysis of fresh tissue and the DIEP procedure scheduling at the Huntsman Cancer Institute implicated a maximum recruiting cap of two participants per week. Our minimum recruiting threshold was ten participants who received the same PSP application. As the main outcome of this study was detecting surviving bioburden, some homogeneity within a small participant cohort is acceptable, even if the bioburden numbers may not be broadly applicable to all patients.

While modern surgeries exhibit far fewer infections than in the past, PSP-surviving skin flora remains an SSI risk. As bioburden was present in the tissue of every patient sampled, additional skin disinfection practices that can be utilized before clinical PSP is performed merit future investigation. Antiseptic diffusion may be limited by the time frame of PSP, the available concentration, and the diffusion characteristics of clinical antiseptics today. Testing current and new PSP technology using full-thickness skin sampling to detect surviving skin flora may lead to improved antiseptic strategies to increase decolonization efficacy. Having observed that skin flora survives PSP similarly in human and pig skin, our previously established pig model may be an effective translational system to test current and develop future PSP technologies [23,24]. We encourage researchers to apply the tissue blend method during antiseptic product development to account for deep-dwelling bacteria and more thoroughly resolve organisms that increase SSI risk.

## 5. Conclusions

Current PSP approaches do not create a sterile field as they leave viable skin-dwelling bacteria on patient skin. Destructive skin testing techniques that incorporate large surface areas and full-thickness excisions may be more effective at resolving bacterial PSP survivors that contribute to SSI risk. New PSP approaches may be needed to overcome residual bioburden and should be tested using destructive sampling. Based on the clinical translatability observed from this study to our established porcine model, we propose that testing the efficacy of improved PSP technologies using the tissue blend method in our porcine model may lead to reduced infection risk for millions of patients.

## Data Availability

The original contributions presented in the study are included in the article, further inquiries can be directed to the corresponding authors.

## Abbreviations

The following abbreviations are used in this manuscript:

PSP	Preoperative Skin Preparation
SSI	Surgical Site Infection
US	United States
CFU	Colony Forming Units
ASTM	American Society for Testing and Materials
CHG	Chlorhexidine Gluconate
DIEP	Deep inferior epigastric perforator artery flap procedure
D/E	Dey–Engley Neutralizing Broth
H&E	Hematoxylin and Eosin
IF	Immunofluorescent
LTA	Lipoteichoic acid
REML	Restricted maximum likelihood
